# A Systematic Review Comparing Digital Subtraction Angiogram With Magnetic Resonance Angiogram Studies in Demonstrating the Angioarchitecture of Cerebral Arteriovenous Malformations

**DOI:** 10.7759/cureus.25803

**Published:** 2022-06-09

**Authors:** Aishwarya Raman, Manish Uprety, Maria Jose Calero, Maria Resah B Villanueva, Narges Joshaghani, Nicole Villa, Omar Badla, Raman Goit, Samia E Saddik, Sarah N Dawood, Ahmad M Rabih, Ahmad Mohammed, Tharun Yadhav Selvamani, Jihan Mostafa

**Affiliations:** 1 Internal Medicine, California Institute of Behavioral Neurosciences & Psychology, Fairfield, USA; 2 Gynecology, California Institute of Behavioral Neurosciences & Psychology, Fairfield, USA; 3 Research, California Institute of Behavioral Neurosciences & Psychology, Fairfield, USA; 4 Psychiatry and Behavioral Sciences, California Institute of Behavioral Neurosciences & Psychology, Fairfield, USA; 5 General Surgery, California Institute of Behavioral Neurosciences & Psychology, Fairfield, USA; 6 Medicine, California Institute of Behavioral Neurosciences & Psychology, Fairfield, USA; 7 Pediatrics, California Institute of Behavioral Neurosciences & Psychology, Fairfield, USA

**Keywords:** asl-mra, tof-mra, brain avm, 4d dsa, swi, mra, dsa, avm

## Abstract

In brain arteriovenous malformations (AVMs), there is mismatched communication between arteries and veins, causing a nidal bed between them. This systematic review explores whether a magnetic resonance angiogram (MRA) can be used as a diagnostic imaging tool instead of a digital subtraction angiogram (DSA).

Utilizing PubMed, Cochrane, and Google Scholar, as well as the Preferred Reporting Items for Systematic Reviews and Meta-Analyses (PRISMA) guidelines for article selection, a literature search was conducted over the past five years.

Eleven studies were included, with a majority of the articles suggesting a potential for consideration. Arterial spin labeling (ASL) versus time-of-flight (TOF) scans was a comparison study, in addition to the study on pseudo-continuous arterial spin labeling (pc-ASL), which proved its high sensitivity in comparison with DSA scans. Other studies included quantitative magnetic resonance angiogram (Q-MRA) measuring the blood flow and susceptibility weighted imaging (SWI) modality. Although promising, digital subtraction angiogram (DSA) scans have diagnostic superiority. In addition, articles discussed follow-up magnetic resonance angiogram (MRA) scans after surgery.

Overall, digital subtraction angiogram remains the gold standard due to its superior spatial resolution and hemodynamic properties; these are the key limitations of magnetic resonance studies. MRA has demonstrated its ability to reproduce high-quality diagnostic images for arteriovenous malformation (AVM) angioarchitecture; however, coupled with their limitations, not many studies with large sample sizes over longer periods have been conducted, and we urge more research into it.

## Introduction and background

Cerebral arteriovenous malformation

Arteriovenous malformations (AVMs) arising in the brain are pathological links formed among arteries and venous via the nidus acting as an intermediary between them. They commonly present as a headache or seizure or could be as serious and fatal as an intracranial hemorrhage [1]. Therefore, management varies from simple observation to stereotactic radiosurgery, resection, and in some cases embolization or combination [2]. Furthermore, all the abovementioned procedures do come with their own potential risk of hemorrhage and seizures, which could lead to death and disability, making it quite an important pathology to understand and implement proper diagnostic imaging to avoid such sinister risks associated [3,4].

Digital subtraction angiogram

This is a type of diagnostic procedure used to visualize very small-sized vasculature ranging in sizes from 4 to 5 millimeters (mm) and as miniature as 1 mm in terms of ophthalmic arteries [5]. Currently, it is the gold standard in terms of visualization and diagnosing angiographic features of the various majority of cerebrovascular pathologies. In recent times, the combined use of two-dimensional (2D) digital subtraction angiogram (DSA) and three-dimensional (3D) DSA is the current practice that helps in the evaluation of the angioarchitecture of arteriovenous malformations [6,7]. Computer-assisted radio monitoring (CARM) and cone-beam computed tomography systems are the methodologies used for developing and measuring volume in the vascular body and are the primary techniques encompassed in four-dimensional (4D) DSA (or time-resolved DSA) [8,9]. The only limitation of digital subtraction angiogram is the contrast-induced procedure and its risk of possessing ionizing radiation; however, the advantages outweigh the risk by a huge margin [10].

Magnetic resonance scans

Acknowledging the advancement in magnetic resonance angiogram (MRA) and its various interpretation in clinical use, there are a couple of studies established on its applications in cerebrovascular malformations. This review includes the following MR sequences.

Non-contrast-enhanced (NCE) 4D dynamic MRA was developed quite recently. Spatial resolution measures from 1 to nearly 2 cubic millimeters (mm^3^), whereas temporal resolution measures up to 100 milliseconds, thereby making it useful for demonstrating hemodynamics in the intracranial blood vessels [11].

In time-of-flight (TOF) scans, arteries are visible as bright entities due to the enhancement of the inflow in arteries, whereas vice versa for the venous drainage due to T2 losses. TOF works by dialing down continuous pulses of radio frequencies, which brings about the reduction of magnetization while demonstrating diagnostic images of stationary brain tissue [12].

Susceptibility weighted image (SWI) differentiates between normal brain tissue and abnormal tissue using a long echo gradient and a completely compensated flow technique. It separates arteries and veins easily, hence evaluating both the blood vessels simultaneously. Paramagnetic susceptibility is noted largely in the breakdown of hemoglobin, in addition to making it a non-contrast study as well. One of the other benefits is its ability to recognize unruptured AVMs [13].

Quantitative MRA (Q-MRA) is fashioned in such a way as to measure intracranial flow in the blood vessels. This is demonstrated by the use of pulse sequences, thereby forming tissue parameters to assess the flow [14].

Arterial spin labeling (ASL) is used to establish blood flow in the brain. It uses the same suggests water proton spin that becomes epithets that are electromagnetic before entering the flow. On reaching the capillaries, the aforementioned epithets act as a tracer in developing microvascular malformations, which in turn leads to perfusion in the cerebral map to be designed. It turns out to be positive due to the absence of intermediary capillary beds in AVM due to the formation of a shunt by transmitting the signal, which, however, under normal circumstances, would have not been possible due to T1 decay being less than the transit time in the capillary. Some scanners, however, cannot maintain frequency that long and hence need adjustments in the amplifier; hence, the pseudo-continuous arterial spin labeling (pc-ASL) method is incorporated where a long singular pulse replaces the previous one [15-17].

## Review

The need for a systematic review

While further researching on this, few studies showcased that MRA in conjunct use with DSA was beneficial in diagnosing micro-AVMs. The answer to whether the use of MR studies has superseded cerebral angiography yet remains unclear due to the superiority of DSA in spatial resolution and in the recognition of draining veins, as well as still considering this scan as the gold standard.

Therefore, the primary purpose of this paper emphasizes exploring the association between the use of MR studies and various advances in this imaging study given its noninvasive approach, which the conventional scans lack. Furthermore, this study attempts to clarify whether MR scans could be considered in diagnosing AVM, including microvascular malformations, instead of the age-old DSA.

Methods

The main databases used in this study for literature search are PubMed, Cochrane, ScienceDirect, and Google Scholar. In addition, we adapted the Preferred Reporting Items for Systematic Reviews and Meta-Analyses (PRISMA) guidelines. The search was performed on March 8, 2022, using key phrases “cerebral arteriovenous malformation OR brain AVM OR AVM, AND DSA OR 4D DSA OR digital subtractive angiography AND MRA OR MR scan OR TOF-MR OR ASL-MR.”

Different types of studies, including those in which patients were scanned with different MR studies along with DSA or separately using the scans, were included in our search. Using the MeSH keywords and phrases (“intracranial arteriovenous malformations/diagnostic imaging” (MeSH) OR “intracranial arteriovenous malformations/radiotherapy” (MeSH)) AND (“magnetic resonance angiography/methods” (MeSH) OR “magnetic resonance angiography/therapeutic use” (MeSH) OR “magnetic resonance angiography/trends” (MeSH)) AND (“angiography, digital subtraction/methods” (MeSH) OR “angiography, digital subtraction/therapeutic use” (MeSH) OR “angiography, digital subtraction/trends” (MeSH)), 21 studies that are relevant were found in the database of PubMed.

However, before the application of criteria for any exclusion or inclusion, a total of 1305 studies were found on PubMed. Removal of duplicates, revisiting of abstracts, and literature title application of inclusion criteria of studies published within five years were performed for final article selection. In addition, after the application of the Assessment of Multiple Systematic Reviews (AMSTAR) checklist and CAse REport (CARE) guidelines, the studies amounted to 11.

**Figure 1:**
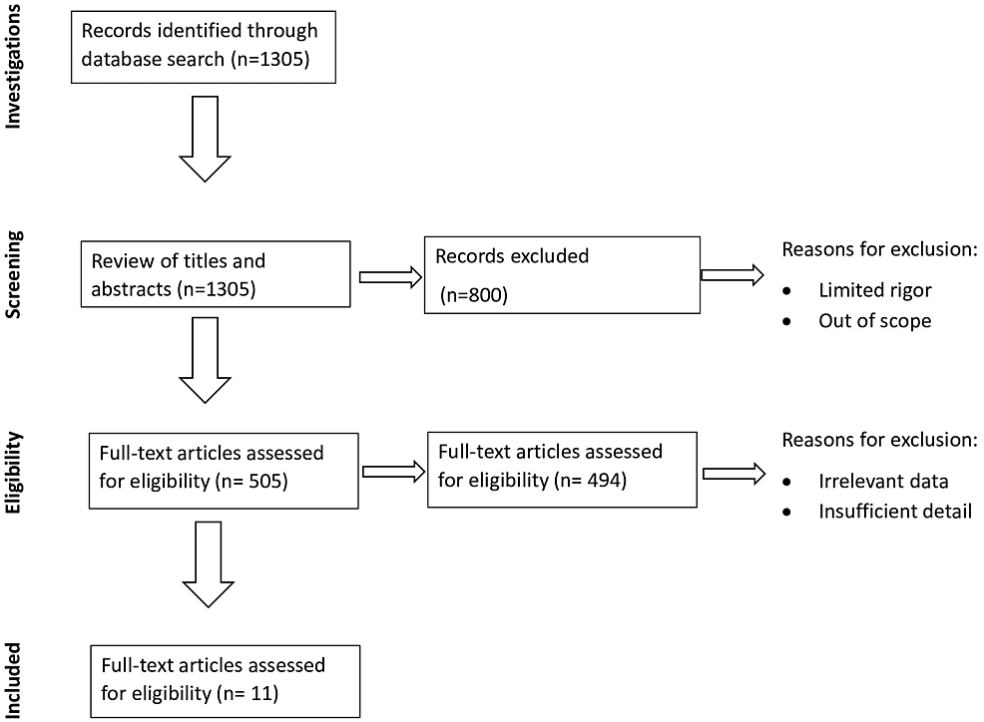
PRISMA flow diagram detailing the study selection process

Figure 1 shows the PRISMA flow diagram detailing the study selection process.

Results

A total of 11 studies that involved various kinds of studies demonstrating the ability of either a type of MRA or DSA in the proper depiction of the characteristics of AVM were correlated.

There are a couple of studies establishing why DSA is considered the gold standard over other noninvasive methodologies [8]. The advancement of 2D and 3D DSA into 4D DSA has nullified the disadvantages both had and has proven to be much more superior in the depiction of the architecture of AVM.

One of the studies compared MR-DSA with conventional DSA and showed very similar imaging of the AVM characteristics [18], whereas another study suggested that SWI can be considered for further AVM evaluation given its higher sensitivities in comparison with DSA [19].

Furthermore, other studies showcase other sequences. For instance, one study established the importance of quantifying the flow in the blood vessels using quantitative MRA [20]. Another study compared and contrasted arterial spin labeling versus TOF and concluded that silent MRA that consists of ASL is better at capturing the angioarchitecture [13]. Finally, one study conducted a retrospective MR-DSA study [8].

In addition to the abovementioned ASL, a more developed version of it, known as pc-ASL, establishes its high sensitivities in evaluating the blood flow, feeding arteries, and draining veins, as well as the measurement of nidal size [21].

Furthermore, a prospective study exhibited the likelihood of 4D dynamic MRA and discussed its benefits and disadvantages, clearly mentioning yet again the chance of this particular type of scan for the angioarchitecture of AVM [22]. Moreover, there is a literature review and case reports signifying conjunct usage and also emphasizing the particular aspects of silent MRA scan and how closely it establishes AVM features [23].

Last, but not the least, a retrospective study was conducted on the gold standard DSA with its recent advancement in 4D or time-resolved scan for surgical planning [24].

On the topic of SWI, there is a later study that highlights the possibility of it being the choice of scan for follow-up patients post-radiotherapy [25].

The characteristics of the studies used for this review are included in ​Table 1.

**Table 1:** Description of the studies that met the inclusion criteria for this review ASL: arterial spin labeling, 7T: 7-Tesla magnetic resonance scan, 3T: 3-Tesla magnetic resonance scan, SOS angle: stack-of-stars angle, MRA: magnetic resonance angiogram, TOF: time-of-flight scans, AVM: arteriovenous malformation, DSA: digital subtraction angiogram, SWI: susceptibility weighted imaging, Q-MRA: quantitative magnetic resonance angiogram, TT: time density time, pc-ASL: pseudo-continuous arterial spin labeling

Author	Purpose of the study	Study type	Sample size	Main findings
Cong et al. [11]	Arterial spin labeling-based 4D MRA scan compared in 3T and 7T	Prospective	14	Demonstration of ASL-based 4D MRA in 7T scan with both Cartesian and SOS angle. Also, a comparison of 3T and 7T with regard to 4D MRA was made, suggestive of the superiority of 7T studies.
Arai et al. [15]	Time-of-flight versus silent MRA scan	Retrospective	29	Comparative study between MRA TOF versus silent MRA for patients diagnosed with AVM. Silent MRA was very efficient in visualizing AVM nidus. Also, TOF-MRA was significantly low by 41.3% in comparison with the former based on Spetzler-Martin’s AVM grading.
Cuong et al. [18]	MRI DSA in comparison with conventional DSA	Retrospective	14	MRI DSA, a noninvasive procedure in locating the AVM compared with traditional DSA, is relatively similar (100%) to the conventional scans.
Wu et al. [19]	Comparison of SWI sequencing in MR studies with gold standard scan	Retrospective	120	In cases of unruptured AVM, it showed that DSA does not play a key role. However, SWI sequencing in MRA illustrated draining veins and other architecture of AVM (e.g., deep venous draining, ectasia in veins, varices of the veins, and associated aneurysms, which were 1, 0.93, 0.94, and 0.83, respectively). Also, 48.15% of silent intralesional microhemorrhages were detected by this MRA sequence.
Brunozzi et al. [20]	Modality of quantitative MRA to diagnose AVM	Retrospective	28	Time density time on Q-MRA correlates with DSA scanning modalities. TT could be used in AVM flow rate as an inversely proportional factor, and more information on the characteristics of AVM, venous stenosis, and rupturing risk could be established by the technique.
Schubert et al. [21]	Comparing pseudo-ASL scan with other MRA scans	Retrospective	32 (8 AVM)	pc-ASL with radial acquisition 3D in comparison with other MRA sequences could have a potential for the characterization of AVM as their sensitivities and specificity are equal with the latter or, in some cases, even higher.
Sandoval-Garcia et al. [8]	Development of DSA	Retrospective comparative study	26 (8 AVM)	2D DSA with concurrent use of 3D DSA for diagnosing AVM features; however, the vascular overlap was its major disadvantage. This is where 4D DSA overcomes such limitations and also helps in the reduction of contrast used for the procedure and the procedure time and thereby reduction in radiation time for the physician.
Lang et al. [22]	Advancement of DSA and comparison with MRA studies	Retrospective	26	Comparing current 2D DSA and 3D DSA with 4D DSA in terms of multiple draining vessels, which the former fails to recognize, causing vascular overlap. Nidal size and blood vessel diameters are the other limitations. DSA versus MRA studies showcase how MRA fails in comprehending tiny blood vessels that are vital in AVM diagnosis. Ferromagnetic implants bring one of the restrictive factors of MRA scans.
Moon et al. [23]	Arterial spin labeling with conventional DSA	Literature review	1	Silent MRA including the arterial spin labeling is the main focus of identifying AVM blood flow. Also mentions its advantages over TOF-MRA. This study concluded by saying that this scan, being a noninvasive one, could be used for AVM diagnosis given its high sensitivity in comparison with conventional DSA.
Chen et al. [24]	DSA in comparison with MRA post-surgery	Retrospective	20	This study focuses on patients who have been scanned with time-resolved DSA post-surgery. This scan shows superior data in comparison with conventional MR studies in identifying the volume of the remnant nidus of AVM.
Finitsis et al. [25]	Post-surgical use of SWI	Prospective	26	SWI as a follow-up scan demonstrates its high sensitivities of approximately 80% and specificity around the range of 88.9% to 95%.

Discussion

Various MR Studies

A prospective study included eight patients with AVM and six in the control group. The images were evaluated by two neuroradiologists using a four-point scale. A Wilcoxon signed-rank test was established to compare 3-Tesla (3T) and 7-Tesla (7T) scans. Both techniques (Cartesian and stack-of-stars (SOS) golden angle radial acquisition) were demonstrated. The results suggested that 7T scans, especially the later technique, were superior with regard to the draining veins present in the AVM, in addition to the delineation characteristics of AVM; not to mention, there was no significant statistical difference between the two. Furthermore, the scanning time was significantly reduced during the radial acquisition in the normal subjects [11].

In total, 29 patients were included, who were diagnosed with AVM either via CT angiogram or conventional DSA. Of these patients, 28 were registered for MRA time-of-flight (TOF) and silent MRA scanning. The study focused on comparing both the MR methodology and suggested that silent MRA could pick up 100% of cerebral AVMs and the nidus was very well visualized. On the other hand, TOF-MRA could only identify 23 out of 28 and less significantly visualize the nidus. Based on Spetzler-Martin’s grade accuracy, silent MRA was 41.3% higher than TOF. Hence, the study concluded by emphasizing that various types of MRA could very well identify the components of AVM, encompassing micro-AVMs as well [15].

Another retrospective study was performed by Cuong et al. at Hanoi Medical University Hospital for 18 months (May 2016 to November 2017) with 20 subjects. Of the 20 patients, six were opted out due to their poor clinical conditions. MRI DSA was done on the remaining 14 pre-procedure. The average age group ranged at approximately 37.4 years, with eight females and six males. All the scans were performed within 10 days of the brain injury and revealed the results to be relatively similar (100%) in locating the nidus of the AVM in comparison with the traditional DSA. Furthermore, the study also suggested that MRI DSA is a promising tool and could be used along with conventional DSA; however, it still needs to be improved in terms of resolution that could be achieved by involving higher resolution scans, for instance, 3T MRI, and increasing the contrast [18].

In unruptured brain AVMs, DSA does not play a major role in assessment and management per se. One of the studies included eight patients (out of 120) who presented with unruptured AVM and had a series of MRI scans. Susceptibility weighted imaging sequencing, which is part of MRI and is also noninvasive, was used for the assessment. The result of the study showed that all lesions were identified by SWI sequencing. It also included exclusively deep venous drainage, venous ectasia, venous varices, and the presence of associated aneurysms on SWI, which were 1, 0.93, 0.94, and 0.83, respectively. Silent intralesional microhemorrhages were detected in 39 (48.15%) patients on SWI, and no significant difference (p > 0.05) was found in angioarchitecture features between patients with and without silent microhemorrhage. Hence, this study suggested that SWI could be potentially used for assessment as it proves well enough to pick up on the angioarchitecture similar to the conventional DSA [19].

A retrospective study over the seven years suggested the role of the hemodynamics of AVM flow between DSA and quantitative MRA. Keeping a keen note on the draining veins close to the nidus, time density time (TT) was correlated to AVM flow, and hemodynamical changes were included as well. TT is defined as the contrast amounting to intensify imaging from 10% to 100% and the reverse with a middle ground of 25% each. Moreover, Q-MRA was used simultaneously for intracranial blood flow measurements to make notes of any hemorrhages and various attributes of the vascular malformations [20].

**Figure 2:**
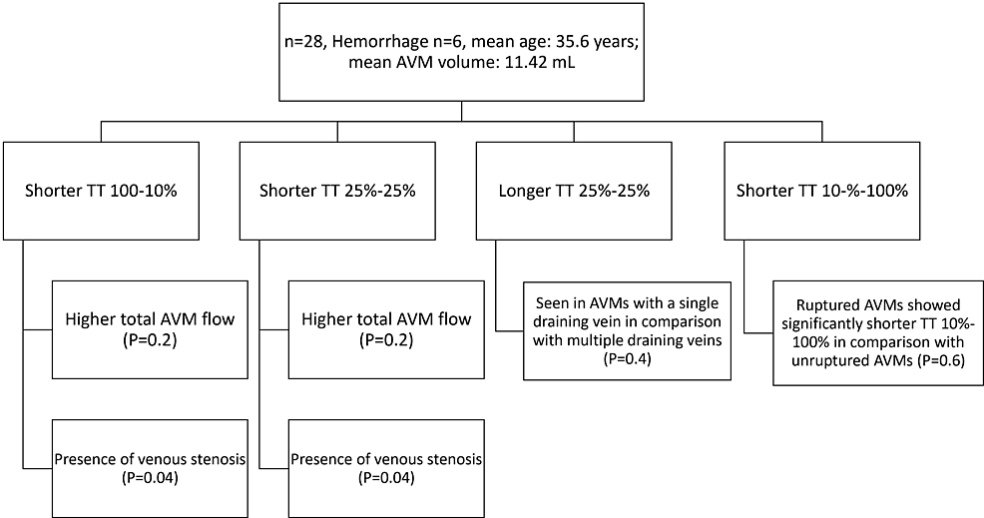
Retrospective study on quantitative MRA imaging scan reporting the time density time (TT) and its subcategories in comparison to AVM hemodynamics TT: time density time, AVM: arteriovenous malformation

A retrospective study on quantitative MRA imaging scan reported the time density time (TT) and its subcategories, comparing this result to AVM hemodynamics, as shown in Figure 2.

The study concluded that time density time could be considered as a measure of the characterization of brain vascular malformations as it is proven to be inversely proportional to the rate of flow in AVM and also demonstrates bleeding potential and resistance to the outflow, as well as stenosis in the veins, via Q-MRA. All the above markers are important in the diagnosis of brain AVM [20].

A study showcased how pseudo-continuous arterial spin labeling (pc-ALS) is a non-contrast and safer study in the evaluation of cerebral vasculature pathologies. It included 32 patients; 12 of these patients presented with AV shunts, out of which eight have AVM and four have arteriovenous fistulas (AVFs); the remaining patients have vasculopathies. All patients were subjected to 2D DSA as the standard in comparison to MRI sequences. This conventional method is used as a standard in many studies due to its better visualization of small AVMs, which other noninvasive technologies fail to pick up, especially clinical MRA, including TOF-MRA and dynamic MRI. Hence, it was assessed whether pc-ALS could help identify cerebral malformations. In comparison, 12 out of 12 were identified by one of the three observers in the study while using pc-ALS. The category included feeding arteries, draining veins, and the size of the lesion. This study included previous studies that showed the superiority of AVM diagnosis by ALS technique and pc-ALS; the sensitivity and specificity of pc-ALS were 100% and 100%, and 91.7% and 100%, respectively, according to readers 1 and 2 of the study [21].

Why Does DSA Remain on Top of Other Scans?

In the single-center, retrospective study conducted by Sandoval-Garcia et al., which took place over 21 months, with three fellowship-trained neurointerventionalists, 26 patients were included, of which eight were healthy individuals, 15 have other correlated pathologies including AVFs/AVMs and aneurysm, and three have vascular stenosis/occlusions. This 2017 study mainly focused on 4D DSA comparison with the other two scans, 2D and 2D DSA [8]. Statistically proven, the precise identification of stenosis in veins, AVFs, aneurysms, feeding arteries, and draining veins were better visualized with 4D DSA in comparison with its counterparts. Unfortunately, 3D DSA faced problems while viewing the nidal characteristics. This could be improved in terms of combinations of temporal and spatial resolution that are very much present in 4D DSA. Also, this had a better evaluation of nidal architecture, as well as fistula visualization, with much improvement if a time-resolved scan was implemented. The limitation of the study was its small sample size. The study suggested, however, that 2D DSA in combination with 3D DSA is used in the major diagnosis of malformation pathology in the brain. They, however, lack overlap of vascular structure identification where 4D DSA is quite useful, in addition to the reduction of radiation onto the physician and even the contrast dose and the most important being reduction in the time frame of the procedure [22].

The study of Sandoval-Garcia et al. acknowledged 4D DSA in a similar manner as other studies mentioned in this article, including superior visualization of perforator arteries and draining veins. Two-dimensional images that are produced by these 4D DSA (time-resolved DSA) scans are also improved in terms of nidal size, blood vessel diameters, and other angioarchitecture features of cerebral AVM. It also shed light on the limitations of the 2D scan, which includes visualization in extreme angulation, and the 3D scan, which fails in terms of temporal information and vascular overlap. It tends to happen in cases of AVMs and AVFs that are found together. This study further mentioned previous literature on the qualitative evaluation of 4D DSA by the author mentioned in the above study (Sandoval-Garcia) in his previous studies. More importantly, it established why MR angiographic studies fail in comparison to the gold standard of current practice. The former still lacks in comprehending tiny blood vessels as they are a vital part of the AVM diagnosis. Moreover, ferromagnetic implants and other restrictive factors could lead to poor diagnostic images. This literature concluded by mentioning a bit about CT DSA, which is considered superior even to MR studies but shares the same limitations [8].

Both Being Used for Conjunct Diagnosis

This literature review also highlights the case report of an incidental finding of AVM while trying to rule out other causes of headache in a 60-year-old female. The study dug deep into the different sequences used in silent MRA. Arterial spin labeling is primarily used in signaling changes in the mid-flow and pulse preparation. In addition, time to echo (TE) is significant in the point-out labeled flow in the blood as it lowers the dispersion phase noted in the voxel space and reduction in susceptibility as well. The final image is the product of the subtraction of the labeled image and the control image. The advantage of silent MRA in detecting slow flow in the blood vessels over TOF MRA was also emphasized. Moreover, this advantage is present in all directions rather than in a single entity. This study concluded that not much research has been conducted in the past on MRA techniques, which have been noninvasive and proved their correlation with conventional DSA scans. The study suggested further research and also consideration of using the former in picturing AVMs [23].

Post-surgery

A retrospective study included 12 patients diagnosed with AVM in a group of 20, with the remaining having other intracranial abnormalities. The mean age group of the patients in this study was 47.5 years. This study was focused on radiosurgery planning. A contrast agent (Omnipaque 300) was used. The present trend suggests the use of 2D DSA with stereotactic MR imaging for management. It also helps if the patient presents with small and residual AVM post-radiosurgery. In previous smaller studies where 3D rotational angiography was used, hemodynamics and temporal information were limited. The current practice is considered optimal. On the other hand, 4D DSA, which is also known as fully time-resolved 3D DSA, is extremely good for the depiction of the vasculature, with better spatial resolution and better temporal imaging, in comparison to the formerly mentioned techniques. Moreover, it is excellent in identifying multiple arteries being fed to a large AVM nidus, which remains one of the lacking entities in 2D DSA and subsequent MR studies. Conventional MR studies are inferior in recognition, and even with advancement, there are errors noted in the precision of volume of the nidus. In conclusion, this study mentioned how time-resolved 3D DSA is far superior in terms of temporal and spatial detailing in addition to the added benefit of identifying feeding arteries that may be multiple in some cases that is in connection with the arteries, thereby suggesting it as one of the feasible options for planning radiosurgery [24].

A prospective study was conducted from March 2012 to March 2018 at the University Hospital of Nancy, France. Susceptibility-weighted angiography (SWAN) showcased higher potential in identifying small malformations and shunts with a slow flow. In comparison with standard DSA, the specificity, sensitivity, and positive and negative predictive values all remained at 85.7%. Although hemosiderin was deposited in the majority of AVMs, it did not interfere with the diagnosis of AVM characteristics in the presence of oil-based contrast used for embolization (n-BCA). It still showcased a high signal in the venous flow. By this, we mean that SWAN was developed based on oxygen evaluation in the shunt as, in the case of AVM, there would be a reduced supply of oxygen from the artery to the vein, thereby making it hyperintense on the imaging, which would normally be hypointense without any oxygen changes. Velocities, whether high or low, worked as TOF effect in SWAN at 1.5 T and 3 T and shifted in the paramagnetic field, respectively. In comparison with previous studies that included other MR modalities (3D TOF and T1), the sensitivities ranged from 76.7% to 84.9%, and specificities from 88.9% to 95%. The main limitations include the inability to tell if there is an obliteration in the case of residual AVM. The other limitations noted in the studies were the false positives and negatives that were the extremely small size of the nidus (in millimeters) and errors in identifying occlusion levels in the arteries, which might lead to hypersignal, leading to considering it as draining veins. On the other hand, SWAN is a non-contrast method and is superior in terms of detailing of spatial diameter in comparison to voxel normal vein. However, this literature concluded with a warrant for larger studies to rule out further errors and mentioned that SWAN could have the potential as a follow-up scan rather than a DSA [25].

Limitations

The primary weakness noted in this study included a lack of adequate sample size, and hence, meta-analysis would not hold any benefit. Assessment of a larger sample size over a longer period would lead to a more beneficial study and increased accuracy. Other limitations are the absence of randomized control trials in this area of research.

## Conclusions

Overall, DSA remains the gold standard due to its superior spatial resolution and hemodynamic properties; these are the key limitations of MR studies. However, more research is warranted on whether MR studies could solely be used as diagnostic imaging for the diagnosis of AVMs in terms of the replacement of the current gold standard being DSA. Many studies have mentioned the conjunct use of both for the diagnosis, and some even mentioned various MR imaging to be quite similar to the invasive conventional scans produced. Moreover, DSA wins in the aspect of spatial resolution, identifying feeding arteries, and hemodynamics as other non-contrast and noninvasive diagnosing imagings have inferior imaging modality. Lastly, this study concludes by emphasizing conducting research with larger study groups within long periods of time for a better understanding of the advantages and disadvantages of MRA.
